# Retrievals and simulations of terrestrial water storage changes and runoff over the Tibetan Plateau: Challenges and opportunities

**DOI:** 10.1016/j.fmre.2025.11.012

**Published:** 2025-11-24

**Authors:** Xueying Li, Di Long, Bridget R. Scanlon, Louise J. Slater

**Affiliations:** a State Key Laboratory of Hydroscience and Engineering, Department of Hydraulic Engineering, Tsinghua University, Beijing 100084, China; b Bureau of Economic Geology, Jackson School of Geosciences, The University of Texas at Austin, Austin TX 78705, USA; c School of Geography and the Environment, University of Oxford, Oxford OX1 3QY, UK

**Keywords:** Terrestrial water storage change, Runoff, Satellite retrievals, Data-driven and hybrid approaches, Tibetan Plateau

## Abstract

The Tibetan Plateau (TP), often referred to as the “Water Tower of Asia”, supplies freshwater to nearly 2 billion people, yet its water resources are increasingly threatened by climate change. Terrestrial water storage (TWS) and runoff are key indicators of water security, directly influencing downstream water availability and use. In this review, we assess recent advancements in the estimation of TWS change and runoff over the TP, while identifying both challenges and opportunities for future research. We provide a comprehensive summary of recent developments in satellite-based TWS change retrievals, improvements in runoff modeling, and the integration of data-driven and hybrid approaches for TWS change reconstruction and runoff simulation, emphasizing cutting-edge space observational techniques and interdisciplinary methodologies. We highlight major challenges in the retrieval and simulation of TWS change and runoff, including limited in-situ data, coarse spatial resolution of TWS change observations, the complexity of hydrologic processes that challenge machine learning applications, and the risk of equifinality due to inadequate model calibration. Furthermore, we explore strategies for overcoming these challenges, with a particular focus on the integration of multisource datasets and hybrid modeling approaches. This review aims to offer valuable insights into the estimation of water storage changes and runoff over the TP. The approaches discussed are not only crucial for understanding hydrologic responses to climate change but also essential for informing adaptive water management strategies in this vulnerable high-mountain region.

## 1 Introduction

The Tibetan Plateau (TP), often referred to as the “Water Tower of Asia”, plays a vital role in providing freshwater to nearly 2 billion people across the continent [1]. With an average elevation of over 4000 m, the hydrologic processes of the TP remain relatively unaffected by human activities, yet they are highly sensitive to climate change. Terrestrial water storage (TWS) and runoff are two critical determinants of regional available water resources for sustainable development. Although TWS encompasses all forms of water storage, including the static water stored in rivers, the contribution of river water storage to TWS variability is generally minor, compared with other major components such as glacier mass, lake water, soil moisture, and groundwater storage. This is because river channels typically have small storage capacities relative to the surrounding terrestrial and subsurface storage. Runoff or river discharge, on the other hand, represents the dynamic component of the river system and reflects the rate of water transport through the drainage network rather than the storage itself. While river storage and runoff are physically interconnected, since changes in storage influence discharge and vice versa, their temporal scales and hydrological implications differ. Therefore, TWS is a state variable indicating the system’s buffering capacity, whereas runoff is a flux variable that represents the immediate and readily renewable freshwater. These two factors are directly tied to water availability and supply capacity of the TP [2,3], exerting profound impacts on the large populations living in the downstream regions [4,5]. Published studies have indicated that shrinking glaciers of the TP have protected large populations from drought stress by providing meltwater during hot/dry years [6], and runoff changes of major TP’s rivers are particularly important to downstream agricultural irrigation [4,5,7] and hydraulic engineering [8].

The unique high-elevation terrain and atmospheric circulation of the TP, influenced by monsoon patterns and upper-level westerly winds, combine to generate the region’s freshwater resources [9]. TWS and runoff are complementary and physically interconnected, representing the key linkage between climate change/variability and hydrologic responses over the TP. According to the 2024 scientific report of China’s Second Tibetan Plateau Scientific Expedition and Research Project (http://www.step.ac.cn/info/15647; in Chinese), the total water storage in this region exceeds 10,000 km^3^, primarily consisting of glaciers, lakes, permafrost, and multi-year mean water storage of major rivers. The most significant climate impact on TP’s TWS change is shown as glacier retreat, lake expansion, and permafrost degradation [1], with significant effects on large-scale atmospheric and energy cycles, as well as direct contributions to meltwater supply for downstream areas. In addition, the TP is the origin of six major transboundary rivers in Asia, i.e., the Amu Darya, Indus, Ganges, Brahmaputra, Salween, and Mekong rivers, feeding two of China’s largest rivers, the Yangtze and Yellow rivers. Runoff originating from the TP constitutes a considerable water resource and exerts a significant influence on water use across Central, South, and Southeast Asia, including the Indo-Gangetic Plain, the Mekong Plain, and the lower reaches of the Yangtze and Yellow rivers. Rivers in China’s southwestern regions, which originate from the southeast TP, have an annual outflow of approximately 580 km^3^. This volume is comparable to 95% of China’s total annual water use in 2020 (610 km^3^) [10]. In addition, runoff from the headwaters of the Indus and Yellow rivers contributes over 30% of the total discharge in their respective basins [11].

Climate warming and changes in atmospheric circulation patterns have profound impacts on both water storage and runoff over the TP. Recent projections indicate that these changes may intensify in the future, threatening the sustainability of water resources across this region [1,3]. Observation-based analyses show a warming rate of 0.44 °C/decade across the TP from 1979 to 2020 [12,13], nearly tripling the previous rate of 0.16 °C/decade observed between 1955 and 1996 [14], and exceeding the global average warming rate of 0.14 °C/decade from 1953 to 2012 [15]. This warming trend has accelerated glacier and snowpack melt, depleting solid water resources and altering the contribution of meltwater to runoff. Additionally, since the 1980s, shifts in the monsoon-westerly system, including the weakening of the South Asia Monsoon [16,17] and the strengthening of the westerlies [18], have altered the spatiotemporal distribution of atmospheric water vapor and precipitation over the TP [19]. These shifts in water vapor and precipitation are amplified by climate variability such as a weakening of El Niño during 2003–2017 [20], and further influence changes in water storage and runoff, contributing to the spatial disparity in the region’s water resources [1]. Key contributing factors include substantial depletion of solid water storage in the exorheic (open) TP [21,22], expansion of liquid water storage in the endorheic (closed) TP [[23], [24], [25]], and significant changes in runoff and related components [[26], [27], [28]]. These changes not only directly influence water supply for downstream areas [3,4,29], but also are inherently associated with critical water security issues over the TP and surrounding areas, such as the retrogressive thaw slumps due to the thawing of ice-rich permafrost [30,31] and flood risks caused by collapsing glaciers [32]. Furthermore, the significant loss of solid water resources over the Pamir and Himalayan regions is projected to cause irreversible declines in TWS under a mid-range carbon emissions scenario by the mid-21st century [3]. Although runoff is projected to increase for seven exorheic rivers on the TP at the end of the 21st century, primarily due to increased precipitation and meltwater, this runoff increase will not reduce the population fraction living under water scarcity conditions in the Indus and Ganges basins based on population projections [33]. These future projections further underscore the need for an integrated understanding of changes in TWS and runoff over the TP, which is crucial for evaluating water resource sustainability and future risks.

Despite the TP’s high vulnerability to climate change, research on changes in TWS and runoff remains limited due to the challenges of data availability and model performance in such a complex terrain and climate system. The TP is one of the most data-scarce regions globally, characterized by extreme conditions that hinder direct observations. As a result, hydrologic models are heavily relied upon for water cycle analysis in this region. However, many global models fail to accurately simulate critical processes related to glaciers and groundwater, leading to significant underestimations of TWS trends [34]. Moreover, the heavy reliance of hydrologic models on runoff parameters, which are typically calibrated with observed streamflow [35], further complicates the extrapolation of information from gauged to ungauged or poorly gauged basins [36,37]. These limitations highlight the urgent need for new observational technology and interdisciplinary approaches to improve the estimation of TWS changes and runoff over the TP.

In this review, we summarize recent advancements in the estimation of water storage changes and runoff over the TP, while identifying key challenges and opportunities for future research. Specifically, we examine the use of multisource remote sensing data to retrieve changes in TWS and water storage components, the application of machine learning techniques to reconstruct hydrologic information, and recent developments in runoff simulation using both hydrologic models and data-driven approaches. We discuss the major challenges in retrieving and simulating TWS changes and runoff over the TP, including the coarse resolution of TWS change observations, limitations of data-driven approaches in interpreting complex hydrologic processes, and the risk of equifinality in hydrologic model calibration, where multiple parameter sets can equally fit the data [38,39]. Finally, we present strategies to address these challenges, mainly including the integration of multisource datasets and hybrid process/machine learning models. This review aims to offer new insights into TWS change and runoff dynamics over the TP, from an integrated perspective that combines water storage and hydrologic flux. By considering both TWS and runoff, this review provides a more comprehensive understanding of cryospheric and hydrologic responses to climate change over the TP, with valuable implications for policymakers in formulating adaptive water resources management strategies for this vulnerable high-mountain region.

## 2 Tibetan plateau

**Fig. 1:**
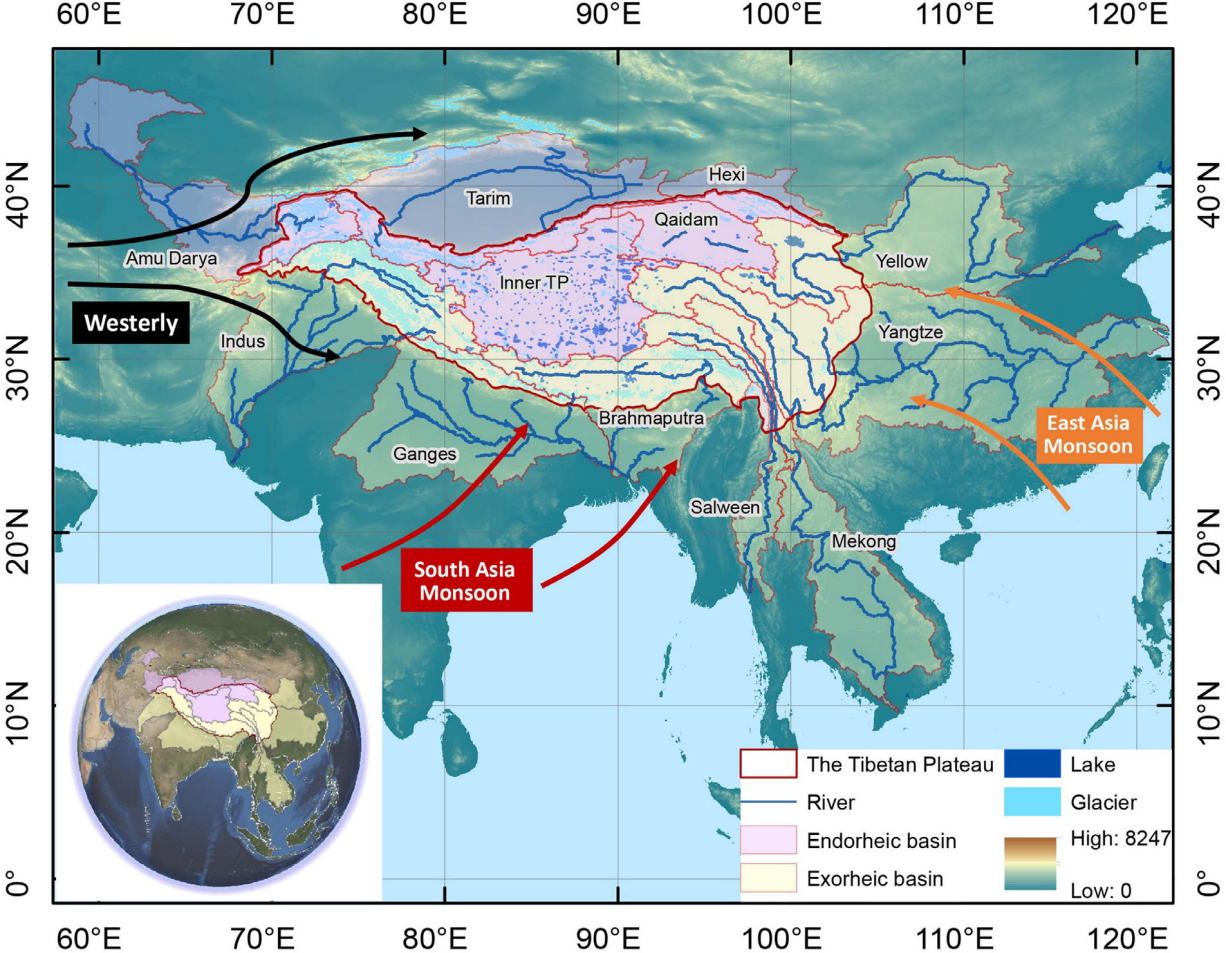
**Lakes, glaciers, and major river basins on the Tibetan Plateau.** This figure is modified from Li et al. [3].

The boundary of the TP (Fig. 1) is defined by the 2500 m elevation contour (68–104°E, 26–40°N) and the river basin boundaries outlined by Zhang et al. [24], based on geographic datasets and digital elevation models (DEMs). The main sources of water vapor over the TP are large-scale vapor transport from the South Asia monsoon, westerlies, and the East Asia monsoon [1]. The South Asia monsoon and mid-latitude westerlies are the dominant atmospheric systems that drive the spatiotemporal variability in water vapor and precipitation, contributing about 77% of the total precipitation on the TP [40]. The South Asian monsoon, which transports moisture from the Indian Ocean, provides significant precipitation to the southern TP, particularly in the Himalayan regions [41]. In contrast, the westerlies, originating from the Mediterranean and Iranian Plateau, predominantly influence precipitation in the western and northern parts of the TP [42]. The interplay between these two systems, via circulation adjustments, further modulates the distribution of water vapor and precipitation across the plateau [1].

Termed “the third pole of earth”, the TP comprises an abundant amount of solid water reservoirs in the form of subsurface ice in permafrost environments, covering a total area of about 1480,000 km^2^ [43], and 77,000 glaciers across the high mountain ranges covering 83,000 km^2^ [10,44]. Additionally, the TP is one of the most densely lake-covered regions in the world, with about 1400 lakes larger than 1 km^2^ covering approximately 50,300 km^2^ [45]. The TP is home to seven exorheic (open) basins (the Indus, Ganges, Brahmaputra, Salween, Mekong, Yangtze, and Yellow River basins) and five endorheic (closed) basins (the Amu Darya, Tarim, Inner Tibetan Plateau, Qaidam, and Hexi basins) (Fig. 1; Table 1). The exorheic basins, located in the southern and eastern parts of the TP, account for about 56% of the plateau’s total area and receive an average annual precipitation of around 900 mm/year (Table 1). These basins are crucial for water supply, particularly due to the significant presence of glaciers in the region. For instance, glaciers in the Hindu Kush−Himalaya Mountains contribute to the water needs of approximately 130 million people downstream in the Indo-Gangetic Plain [4]. Similarly, the Nyainqentanglha Mountains, located in the southeast TP, contain the highest concentration of maritime glaciers on the plateau, where accumulation and melt are largely impacted by the monsoon climate [46]. In contrast, the endorheic basins, found in the central, northern, and western TP, cover 44% of the total area and receive much less precipitation, around 380 mm annually (Table 1). These basins are rich in lakes, which occupy about 73% of the TP’s total lake area [1].

**Table 1:** **Geographical and hydrologic information of the entire Tibetan Plateau and each basin**.

Name	Location range	Elevation (m)	Area (km^2^)	Percentage of area within the TP to the entire basin (%)	Annual mean precipitation (maximum/minimum)	Annual mean temperature (maximum/minimum)
*The entire Tibetan Plateau*						
The TP	68°−104°E, 26°−40°N	408–8247	3066,700	100	624 (678/550)	−2.7 (−1.4/−4.0)
*Exorheic (open) basins*						
Brahmaputra	82–98°E, 27–31°N	408–8247	385,973	59	1016 (1172/902)	0.2 (1.7/−0.8)
Ganges	78–88°E, 27–31°N	547–7982	85,273	9	1371 (1542/1192)	1.9 (3.3/0.2)
Indus	68–82°E, 30–37°N	779–7528	319,695	37	622 (737/528)	−4.8 (−3.0/−6.6)
Mekong	94–100°E, 26–34°N	1403–5983	90,802	12	797 (918/633)	0.6 (1.7/−0.7)
Salween	91–99°E, 26–33°N	859–6382	111,102	42	807 (912/690)	−1.1 (0.3/−2.6)
Yangtze	91–105°E, 27–36°N	781–6317	482,063	25	874 (1016/761)	0.1 (1.0/−1.2)
Yellow	96–104°E, 32–38°N	1762–6090	249,876	29	661 (786/564)	−1.3 (−0.1/−2.9)
*Endorheic (closed) basins*						
Amu Darya	68–75°E, 35–40°N	934–6935	125,809	20	301 (430/208)	−4.5 (−2.7/−6.2)
Hexi	93–101°E, 38–40°N	1950–5610	61,933	35	418 (524/334)	−5.3 (−4.2/−7.9)
Inner TP	79–94°E, 30–39°N	2854–6317	709,329	100	364 (501/274)	−5.9 (−4.0/−7.9)
Qaidam	90–99°E, 35–39°N	2655–6552	253,434	100	218 (271/164)	−0.5 (0.8/−2.2)
Tarim	73–93°E, 35–40°N	1805–7390	191,411	21	327 (443/253)	−7.5 (−6.0/−8.9)

*Note*: Precipitation and temperature are estimated from gridded data sets of China Meteorological Forcing Dataset v2.0 during the 1981–2020 time period.

**Fig. 2:**
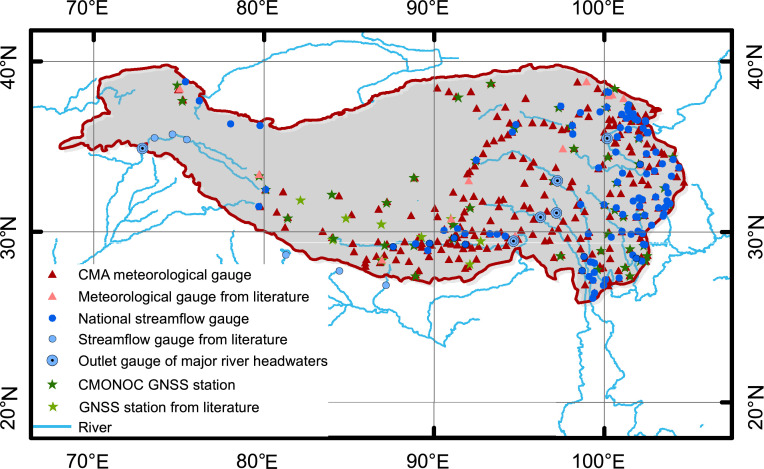
**Ground observation networks over the Tibetan Plateau.** Meteorological gauges, streamflow gauges, and GNSS stations are presented by red triangles, blue circles, and green stars, respectively, with dark colors indicating gauges from the China national observation network and light colors indicating gauges from the literature. Specifically, meteorological gauges were obtained from the China Meteorological Administration (CMA) and Ma et al. [49]. Streamflow gauges were obtained from the Ministry of Water Resources of the People’s Republic of China and Li et al. [50]. Outlet gauges of major river headwaters are marked by a larger circle and center mark, including the Besham Qila, Nuxia, Jiayuqiao, Changdu, Zhimenda, and Tangnaihai gauges for headwaters of the Indus, Brahmaputra, Salween, Mekong, Yangtze, and Yellow rivers, respectively. GNSS stations are obtained from the Crustal Movement Observation Network of China (CMONOC), Wang et al. [51], and Li and Long [52].

**Fig. 3:**
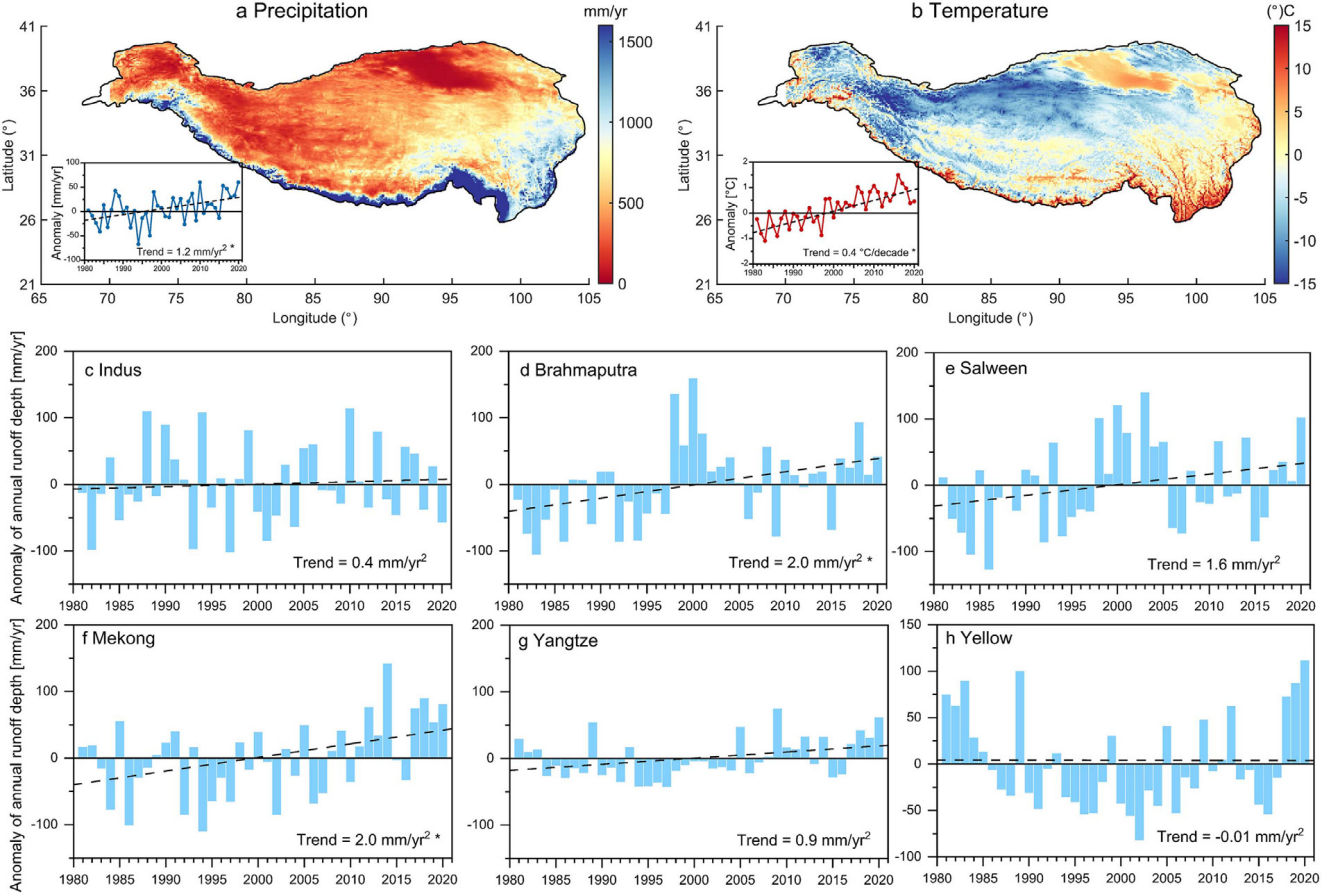
**Changes in meteorological and hydrologic variables over the Tibetan Plateau and major basins.** (a,b) Spatial pattern of annual mean precipitation and near-surface temperature for the 1981–2020 period, with inserted plot showing annual anomaly of the corresponding variable, relative to the climatology of the 30-year mean of 1981–2010. Both precipitation and temperature are estimated from gridded datasets of the China Meteorological Forcing Dataset v2.0. c-h Annual anomaly of runoff depth for the headwaters of the Indus (c), Brahmaputra (d), Salween (e), Mekong (f), Yangtze (g), and Yellow rivers (h). Observed runoff data are obtained from Long et al. [19] and Li et al. [50], corresponding to outlet gauges with the center mark in Fig. 2, and relative to the climatology of the 30-year runoff mean of 1981–2010. The dashed line in each time series indicates the trend for the past 4 decades, with the trend value shown in the right corner. The symbol “*” indicates a significant change (the Mann-Kendall test at a 5% significance level).

Ground observation networks over the TP, including meteorological and streamflow observations for hydrologic research and Global Navigation Satellite System (GNSS) stations for monitoring vertical crustal deformation (a key indicator of ground-based TWS change retrieval), are currently concentrated in the low-elevation southern and eastern part (Fig. 2). However, the lack of gauges over the northern and western TP limits the representative of ground observations, particularly over high-elevation regions. For instance, Immerzeel et al. [47] indicated that in the upper Indus basin (located in the western TP), the amount of precipitation required to sustain the observed mass changes of large glacier systems should be far beyond the precipitation values observed at valley stations (up to twice to 10 times). Based on gridded data sets of China Meteorological Forcing Dataset v2.0, which integrates meteorological observations and reanalysis data, the TP has experienced a significant wetting and warming trend (*p* < 0.05) for the past 4 decades (Fig. 3a–b), with an increase rate at 1.2 mm/year (precipitation) and 0.4 °C/decade (temperature). Both precipitation and temperature show a marked spatial gradient, decreasing from the southeast to the northwest of the TP. In the southeast, the annual average temperature reaches up to 20 °C, with annual precipitation exceeding 1000 mm. In contrast, the northwest experiences annual temperatures below 0 °C and receives <100 mm of precipitation annually [48]. As for runoff changes over major rivers, except for a very slight decreasing trend in the Yellow River basin, all other basins experienced an increasing runoff trend, with a significant increase in the Brahmaputra and Mekong basins (*p* < 0.05).

## 3 Satellite retrievals of water storage changes

The analysis of water storage has received less attention in past studies compared to the estimation of fluxes (e.g., precipitation and runoff). This is partly due to the limited availability of large-scale in-situ storage observations and the inherent complexity in isolating individual storage components from TWS in hydrologic models [34]. The ground GNSS system enables monitoring of vertical crustal deformation with high spatial and temporal resolution, which is a key indicator of retrieving storage changes based on hydro-geodesy approaches like GNSS-Interferometric Reflectometry (GNSS-IR). The foundation of GNSS-inferred TWS changes is based on Green’s function [53,54], the mass load theory describing that the deformation of the solid earth is an elastic response to the change of TWS*.* However, the sparse and unevenly distributed GNSS stations limit the application of GNSS-IR over the TP. Most GNSS stations are located in the low-elevation southern and eastern TP, but only very limited gauges are in the high-elevation western part (Fig. 2). Combining gauge-based GNSS signals with machine learning methods could be an effective way to address this limitation [55,56]. For example, Zhang et al. [55] simulated crustal deformation caused by hydrologic loading and estimated TWS changes on a 0.5° grid cell across the northeastern TP by employing traditional GNSS inversion techniques and the Extreme Gradient Boosting Machine Learning (XGBML) model.

The loading effect is another uncertainty source of GNSS inversion over the TP. Wang et al. [57] illustrated the impacts of crustal thickening over the TP on loading modeling and inversion associated with water storage variation. That is, the inappropriate use of the global Preliminary Reference Earth Model could result in the incomplete recovery of annual hydrology signals inverted from annual deformation data, only recovering 88% of hydrology signals for truncated degrees of 180. Zhang et al. [58] indicated that the commonly used interferometric reflectometry algorithm could cause high uncertainty over permafrost areas, since it does not consider the bias in the seasonal surface elevation changes due to the active layer thawing.

In general, the accuracy of ground-based hydro-geodesy approaches fundamentally relies on the density of GNSS stations, which struggles to represent storage changes at large scales, particularly over the TP with great spatial heterogeneity. There is an important opportunity to take advantage of remote sensing technologies to improve our understanding of water storage dynamics at large scales. Key developments in this area include: (1) the progress in the Gravity Recovery and Climate Experiment (GRACE) and GRACE Follow-On (GRACE-FO) satellite missions, which have provided over 2 decades of reliable data for quantifying changes in TWS; (2) enhancements in satellite altimetry and optical remote sensing, which are critical for assessing changes in surface water storage such as lakes and glaciers; and (3) the development of microwave remote sensing of InSAR (Interferometric Synthetic Aperture Radar) technology, which enables monitoring of ground deformation as a key indicator of estimating permafrost degradation.

### 3.1 Multisource remote sensing for water storage retrievals

#### 3.1.1 GRACE and GRACE-FO missions

The GRACE (April 2002–June 2017) and GRACE-FO (June 2018 to present) missions, which rely on satellite gravimetry, offer an unprecedented means of quantifying TWS changes. GRACE operates with two identical satellites in near-polar orbits (∼450 km above Earth), with the along-track separation within a range of 220 ± 50 km [59] (https://earth.gsfc.nasa.gov/geo/missions/grace). The satellites measure the differential acceleration caused by changes in local mass distribution, which, in turn, leads to variations in the distance between them. These distance fluctuations are used to retrieve the time-variable Earth’s gravity field and track mass changes, including TWS variations. Following the GRACE mission, GRACE-FO, launched in May 2018, continues to provide valuable data on TWS anomalies, with monthly temporal and ∼300 km spatial resolution [59,60]. Several institutions have processed GRACE signals (Table 2). Among them, three official processing centers within the GRACE science data system (GRACE SDS) include the Jet Propulsion Laboratory (JPL), the Center for Space Research (CSR), and the Helmholtz Centre for Geosciences (GeoForschungsZentrum; GFZ), using spherical harmonic solutions. Recent developments, including mascon (mass concentration block) products, have further enhanced GRACE data, with regularization algorithms for CSR products [61] and Community Land Model-derived gain factors for JPL products [62].

**Table 2:** **Institutions of GRACE data processing**.

Institution	References
Astronomical Institute of the University of Bern	Meyer et al. [63]
Center for Space Research	Save et al. [61]
Combination Service for Time-variable Gravity fields	Jäggi et al. [64]
Delft Institute of Earth Observation and Space Systems	Farahani et al. [65]
Faculty of Geosciences and Environmental Engineering, Southwest Jiao-Tong University	Yu et al. [66]
Groupe de Recherche de Geodesie Spatiale	Bruinsma et al. [67]
Helmholtz Centre for Geosciences (GeoForschungsZentrum)	Dahle et al. [68]
Huazhong University of Science and Technology	Zhou et al. [69]
Institute of Geodesy and Geophysics, Chinese Academy of Sciences	Wang et al. [70]
Institute of Geodesy at Graz University of Technology	Kvas et al. [71]
Jet Propulsion Laboratory	Watkins et al. [72]
Leibniz Universität Hannover	Koch et al. [73]
Southwest Petroleum University	Su et al. [74]
Wuhan University	Guo et al. [75]
Tongji University	Chen et al. [76]
Xi’an Research Institute of Surveying and Mapping and Space Star Technology co., LTD	https://icgem.gfz-potsdam.de/sp/03_other/XISM&SSTC/GRACE01

Numerous studies have applied GRACE and GRACE-FO datasets to quantify TWS change across the TP in the last 2 decades. For example, Meng et al. [77] investigated TWS changes across the TP during 2003–2014 based on GRACE retrievals and revealed an increase in TWS in the central and northern TP but a significant decrease in the southern and northwestern TP. Further, Xiang et al. [78] extended the time period until the recent year of 2021, using both GRACE and GRACE-FO data, and found that the largest water loss was in the Nyainqentanglha Mountains and Eastern Himalayas, at rates of −4.92 Gt/year and −4.34 Gt/year during 2002–2021, respectively. Recent studies have attempted to use machine learning approaches to reconstruct long-term TWS changes over multiple decades, such as from the 1940s [79] or 1960s [80]. But most of these reconstruction studies are conducted at the global scale, without a specific focus on cryospheric processes such as glacier melting. Therefore, the accuracy of the long-term reconstructed TWS changes requires more extensive validation over the TP.

In addition to quantifying TWS changes, GRACE data provide key information in understanding hydrologic and cryospheric processes over the TP. Jointly using GRACE retrievals and hydrologic model outputs, it is shown that GRACE-observed mass loss in the southeastern TP was highly related to glacial melting [81], whereas lake changes dominated long-term trends in TWS over the northeastern TP [82]. In addition, accurate TWS change derived from GRACE retrievals is a critical component in the water balance, which contributes to the assessment of other hydrologic fluxes such as evapotranspiration (ET) [83], and further improves the understanding of the hydrologic and energy budget. Across the mountain regions where the signal of TWS variation is dominated by glacier change, GRACE data provide an important supplement to quantify the glacier mass balance. Xiang et al. [84] found that storage changes derived from the GRACE release-6 solution [71] had good agreement with glacier loss based on satellite altimetry-based analysis across major mountains around the TP. Zhao et al. [46] used GRACE-derived TWS changes as ancillary data to fill the data gap between two satellite altimetry missions, to obtain the continuous glacier elevation change over the southeastern TP.

Moreover, advancements in TWS change estimation offer effective constraints that strengthen the simulation capabilities of hydrologic models [85]. This is because water storage changes, such as glacier retreat and snowpack melting, are inherently linked to meltwater over high-elevation headwater basins on the TP. Therefore, Chen et al. [86] developed a two-stage calibration of the runoff simulation, with one step of calibration based on snow information and the other based on GRACE-derived TWS changes. Huang et al. [87] additionally included the calibration of TWS changes in runoff modeling, which improved the performance of simulated runoff, especially for a more reliable runoff component estimation (e.g., rainfall runoff, snowmelt, and glacier melt). Further, Bai et al. [88] proposed a multi-objective calibration scheme based on GRACE-derived TWS changes and streamflow observations, and a single-objective calibration scheme that only used streamflow. The performance of two lumped hydrologic models [89,90] calibrated by these two schemes was compared over 22 basins in China, including the Mekong, Yangtze, and Yellow basins on the TP. They found that relative to the single-objective calibration, the multi-objective calibration provided more reliable TWS changes and ET without significant deterioration in the accuracy of streamflow simulations, highlighting the importance of TWS constraints for a more reliable understanding of various components of hydrologic models.

#### 3.1.2 Satellite altimetry and optical remote sensing

Satellite altimetry and optical remote sensing technologies (e.g., Table 3) have made significant progress in improving the accuracy of estimating changes in surface water storage components, particularly for lakes and glaciers over the TP. These remote sensing techniques are crucial for understanding changes in different components and their contributions to the overall TWS change. For estimating changes in lake storage, a common approach is to integrate the change in lake area with the change in water level [23,[91], [92], [93], [94]]. Satellite optical images (e.g., the series of Landsat and Sentinel-2 images) are the fundamental data sources to provide consistent changes in lake area [95]. The typical framework of retrieving lake area changes includes the pre-processing of satellite images (e.g., geometric and atmospheric correction and cloud masking), calculation of the water-body index (e.g., the Normalized Difference Water Index, NDWI [96]), and threshold selection for separating lake boundaries (e.g., the Otsu algorithm [97]). Based on optical images, the total number of lakes on the TP increased from 1080 in the 1970s to 1424 in 2018 (+32%), with the total lake area expanding from 40,000 km^2^ to 50,000 km^2^ (+25%) [98]. Changes in water level of the TP lakes have been widely monitored through satellite altimetry, including both radar (e.g., CryoSat-2) and laser (e.g., ICESat) missions. Using approaches of waveform retracking, outlier filtering, and geophysical and atmospheric corrections, published studies have shown that the average water level of lakes on the TP increased by approximately 4 m since the 1970s to 2018 [98]. Leveraging multi-sensor satellite altimetry, the recently developed datasets of lake water level changes [99,100] largely improved our understanding of lake dynamics across the TP over the past decades. After obtaining changes in lake areas and water levels separately, the change in lake storage is finally the integral of area over the elevation change.

**Table 3:** **Commonly used satellite altimetry data and optical images for estimating changes in surface water storage over the past 2 decades**.

Mission	Sensor (type)	Duration	Temporal resolution (d)	Spatial resolution (along-track)	Agency
*Satellite altimetry*					
CryoSat-2	SIRAL (radar)	2010−	369(subcycle 30)	0.3 km	ESA
Envisat	RA-2 (radar)	2002−2012	35	3.4 km	ESA
ICESat	GLAS (laser)	2003−2009	183	70 m	NASA
ICESat-2	ATLAS (laser)	2018−	91	17 m	NASA
Jason-1	Poseidon-2 (radar)	2001−2013	10	2–4 km	NASA/CNES
Jason-2	Poseidon-3 (radar)	2008−2019	10	2–4 km	NASA/CNES
Jason-3	Poseidon-3B (radar)	2016−	10	2–4 km	NASA/CNES
Sentinel-3A	SRAL (radar)	2016−	27	300 m	ESA
Sentinel-3B	SRAL (radar)	2018−	27	300 m	ESA
Sentinel-6A	Poseidon-4 (radar)	2020−	10	<10 m	ESA/CNES/NASA
SWOT	Poseidon-3C and KaRIn (radar)	2022−	21	<100 m	NASA/CNES
*Optical images*					
Landsat 7	ETM+	1999−2022	16	30 m	NASA
Landsat 8	OLI	2013−	16	30 m	NASA
Sentinel-2A	MSI	2015−	10 (single); 5 (combined Sentinel-2B)	10 m, 20 m, 60 m	ESA
Sentinel-2B	MSI	2017−	10 (single); 5 (combined Sentinel-2A)	10 m, 20 m, 60 m	ESA

*Note* SIRAL, Synthetic aperture radar (SAR) Interferometric Radar Altimeter; RA, Radar Altimeter; GLAS, Geoscience Laser Altimeter System; ATLAS, Advanced Topographic Laser Altimeter System; SRAL, SAR Radar ALtimeter; KaRIn, Ka-band Radar Interferometer; ETM+, Enhanced Thematic Mapper Plus; OLI, Operational Land Imager; MSI, Multi-Spectral Instrument; ESA, European Space Agency; NASA, National Aeronautics and Space Administration; CNES, Centre National d’Etudes Spatiale.

Integrating data from multiple altimetric missions and optical remote sensing images can be an effective way to obtain high-temporal-resolution lake changes. However, one challenge is the systematic biases that arise due to differences in reference ellipsoids, geoid models, and retracking algorithms. To address this, one step of removing systematic biases in altimetric data is based on the “optical water level” derived from optical images [23], and the other one is integration of GRACE-derived TWS changes and retrievals of other storage components for consistency validation (see Section 3.3). As for bias correction based on the optical water level, it is estimated using regression relationships between lake shoreline changes from optical images and altimetry-based water level variations. The long-term and consistent optical datasets (e.g., Landsat-derived water levels) provide an ideal reference for integrating multisource lake water level retrievals, particularly when satellite altimetry data have limited temporal coverage over multi-decadal periods. In addition, microwave synthetic aperture radar (SAR) images serve as an effective complement to optical methods for obtaining high-temporal-resolution lake changes, owing to their short revisit time and all-weather imaging capability. Recent studies have combined optical and SAR images with machine learning based water body extractions, largely improving the capacity of investigating lake changes from vast satellite images. For example, Ran et al. [101] used deep learning models of CloudNet [102] and LaeNet [103] to extract water bodies from SAR and Landsat optical images to investigate monthly changes in lake area on the TP (>30 km^2^). Luo et al. [104] developed an automatic method for surface water mapping using SAR images and convolutional networks, focusing on seasonal dynamics of surface water over the TP. Field experiments using unmanned aerial vehicle could examine the variation of lake shoreline and further validate changes in lake areas, such as Li et al. [23] employed at Lake Nam Co and Lake Yamzhog Yumco, and Ran et al. [101] conducted at Lake Selin Co. Combining these ground observations with theoretical verification (e.g., uncertainty propagation), the uncertainty of storage changes in 52 large lakes over the TP (≥100 km^2^; accounting for ∼60% of the total TP lake area) was estimated about ±6% [10,23].

In the case of glaciers, glacier mass change, indicating the gain/loss of glacier storage in terms of water equivalent, is a more commonly used term when comparing changes in glacier storage and other water storage components. Mass changes are converted from volume changes using a standard conversion factor (generally 850 kg/m^3^, based on the average glacier density over the TP) [105]. Glacier volume change is estimated by multiplying the area of the glacier (from the Randolph Glacier Inventory, RGI) by the elevation change. Two primary methods are used to estimate glacier elevation changes: (1) geodetic methods using two or more DEMs from different time periods [44,106,107], and (2) satellite altimetry [[108], [109], [110]]. Rapid improvements in satellite archives have enabled accurate large-scale assessments of glacier mass change. For instance, Brun et al. [21] quantified glacier mass changes across 92% of the glacierized area in High Mountain Asia using DEM differences derived from satellite stereo-imagery. Similarly, Hugonnet et al. [44] provided high-resolution surface elevation change data for almost all of Earth’s glaciers, using previously underused satellite archives. Moreover, recent studies have integrated these two approaches to overcome the limitations inherent in using a single method. For example, issues such as inconsistencies in DEMs from different data sources and time periods [111], low spatial sampling of satellite altimetry at low latitudes [46], and cloud cover in satellite altimetric retrievals, particularly for laser data [112,113], have been addressed by combining altimetry, DEMs, and satellite imagery. Zhao et al. [46] demonstrated this approach by generating high-temporal-resolution glacier mass balance data for the southeast TP, integrating ICESat, CryoSat-2, and ICESat-2 data with optical image-derived DEMs to minimize systematic errors. Glaciological observations using mass-balance measurements provide baselines for investigating glacier changes, and the multi-year observations at the Ata, Halong, Parlung 12, and Qianyong Glaciers [18] have confirmed significant glacier retreat from satellite retrievals. Based on in-situ measurements and uncertainty propagation, the uncertainty of glacier storage changes for the entire TP was estimated to be ±20% by Brun et al. [21] using the vast amount of freely available ASTER optical satellite stereo pairs.

#### 3.1.3 Microwave remote sensing

As an active microwave remote sensing technique, multi-temporal InSAR (Interferometric Synthetic Aperture Radar) missions enable monitoring of ground deformation, a key indicator of estimating permafrost degradation. Liu et al. [114] compared characteristics of seasonal deformation in the permafrost area based on leveling measurements, InSAR monitoring, and hydrothermal-data-based simulation results at the Xidatan station on the TP. The 8-year long-term deformation information provided by leveling measurements showed that InSAR retrievals largely outperformed simulation results, indicated by a closer correlation and a more similar deformation pattern with leveling measurements captured by InSAR measurements. In general, the Differential InSAR (D-InSAR) technology is a fundamental approach by differencing interferograms from two SAR acquisitions after removing the topographic phase component. But this method can be affected by space-time incoherence and atmospheric delay, leading to reduced observation accuracy [115]. Therefore, Berardino et al. [116] introduced the Small Baseline Subset (SBAS) technology for time series analysis in InSAR, which deploys multiple master datasets to minimize the effects of spatial-temporal decorrelation. Based on SBAS-InSAR retrievals, Wang et al. [117] monitored ground surface deformation on the northern and southern slopes of the Tanggula Mountains on the TP during 2017–2020, indicating that the average subsidence rate was 9.1 mm/year over subsiding terrain. Zhou et al. [118] quantified the surface deformation in the Wudaoliang region (located in a large continuous permafrost area of the central TP) from 2014 to 2017, showing an annual deformation rate of −10.28 mm/year.

Integrating InSAR observations with statistical or physical model-based analysis provides an enhanced understanding of the physical mechanisms of ground deformation. For instance, Chen et al. [119] developed a permafrost-tailored InSAR approach by incorporating a temperature-integrated ground deformation model to reconstruct the seasonal and long-term deformation. This approach revealed the spatial patterns of seasonal uplift and long-term subsidence in permafrost regions and their correspondence with land surface temperatures. Utilizing this approach to Sentinel-1 data on the vast permafrost regions of about 140,000 km^2^ of the central TP, this study showed widespread seasonal deformation up to 80 mm with a linear subsidence up to 20 mm/year during 2014–2019. A higher linear subsidence was found in regions with high ground ice content and warm permafrost. Chen et al. [120] coupled InSAR processing with independent component analysis (ICA) to isolate seasonal deformation from InSAR time series, improving the understanding of seasonal freezing/thawing processes over permafrost regions on the TP. By applying independent component analysis on Sentinel-1 InSAR measurements, this study showed an increase of seasonal deformation at 0.17 cm/year during 2015–2019 over the central TP. With the accumulation of long-time series data, InSAR retrievals can capture the inter-annual deformation in permafrost regions. Published studies have reported that permafrost regions with a low rate of inter-annual subsidence were mainly distributed in the northeast of the TP and the Beiluhe River, whereas areas with a high rate of inter-annual subsidence were concentrated along the Qinghai-Tibet Highway [115].

Further, the dynamics of permafrost deformation is highly coupled with permafrost-hydrologic processes over the TP, where thawing permafrost increases active layer water content and alters lake and river water balance. Based on the advantages of InSAR technology to retrieve ground deformation, published studies have estimated permafrost active layer water content and the contribution of ground ice melting to rivers and lakes on the TP. Du et al. [121] derived ground surface deformation curves through Sentinel-1 SBAS-InSAR monitoring, and then constructed a water depth-deformation model to estimate permafrost active layer water content in the alpine grassland environment of the central TP. Wang et al. [122] estimated surface deformation of the source region of the Yangtze river on the TP, and utilized the long-term deformation rate to assess water storage in the permafrost active layer. Results indicated widespread ground ice melting with 55% of the region showing subsidence higher than 2.5 mm/year during 2017–2021, and the released water contributes to about 3% of streamflow. In addition, Wang et al. [123] combined satellite altimetry and the SBAR-InSAR technique, and quantified that about 12% of the lake volume increase of Lake Selin Co on the TP was attributed to permafrost degradation during 2017–2020. These studies revealed the capability of InSAR to quantify permafrost freezing/thawing dynamics, spatiotemporal patterns, and interactions with other hydrologic components in vast permafrost areas, providing valuable insights into the water mass balance over the TP.

### 3.2 Challenges in estimating TWS change over the TP

**Fig. 4:**
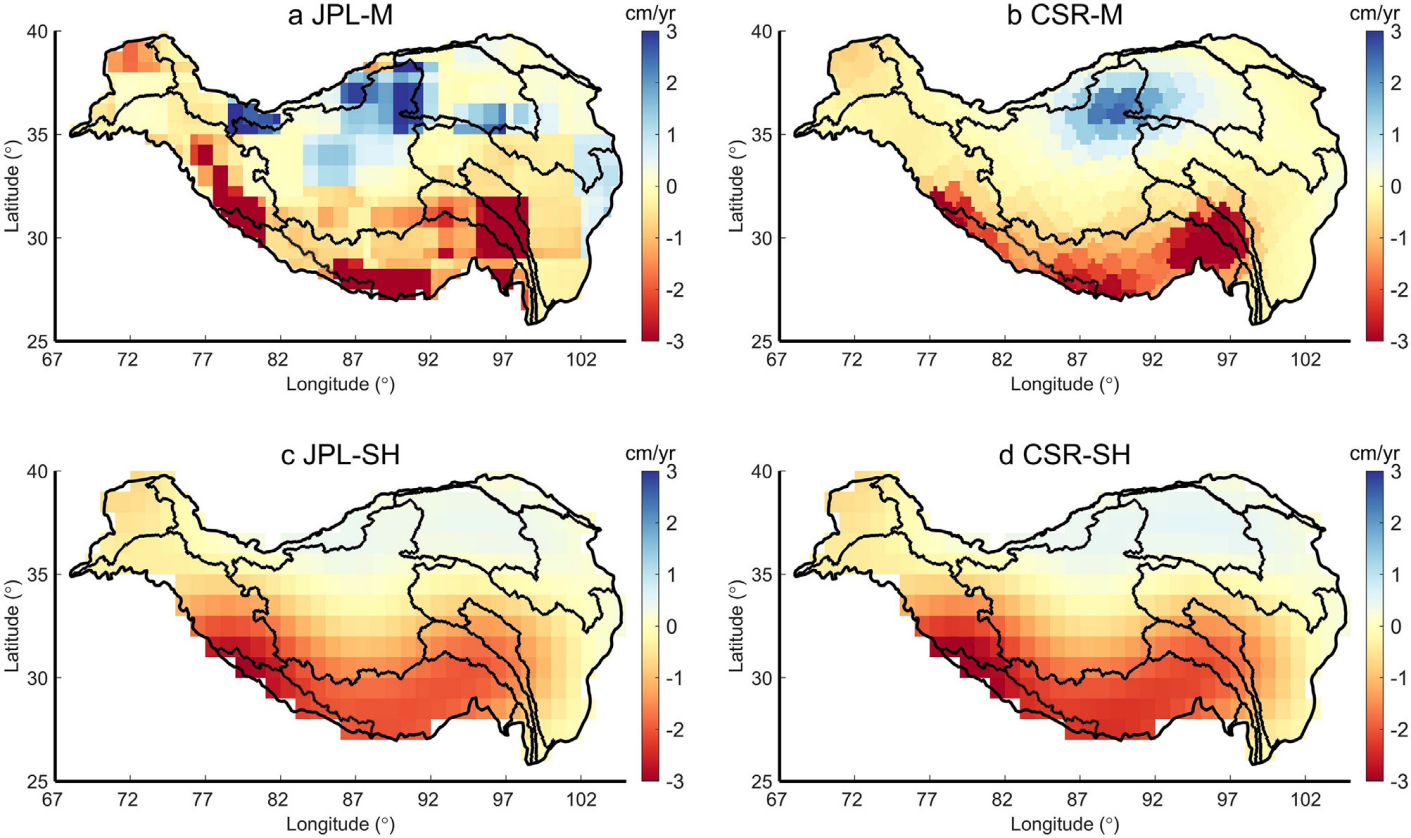
**Trends in TWS over the TP during the GRACE observation period (2002−2017).** Results were derived from four GRACE solutions, including JPL-M (a), CSR-M (b), JPL-SH (c), and CSR-SH (d). Data used to generate this figure were provided by Li et al. [3].

Despite significant progress in analyzing TWS changes using GRACE and GRACE-FO data, several challenges remain due to the inherent limitations of the GRACE mission. One of the primary constraints is the coarse spatial resolution of GRACE retrievals, which is approximately 300 km. The relatively low resolution can result in large uncertainty in TWS change estimation in regions with high spatial heterogeneity like the TP. This is indicated by substantial discrepancies between different GRACE analytical solutions (e.g., spherical harmonic vs. mascon solutions) that are related to filters of removing north-south-oriented stripe features and regularization algorithms of mascons [124,125] (Fig. 4). For example, Jacob et al. [126] used early-stage mascon products to estimate TWS depletion over High Mountain Asia (including the TP and Tien Shan region), at a rate of four Gt/year (4 km^3^/year) from 2003 to 2010. In contrast, Matsuo and Heki [127] found a decrease of up to 47 Gt/year for the High Mountain Asia during 2003−2009, using spherical harmonic solutions. These large discrepancies hinder our understanding of TWS responses to climate change.

Further analysis by Jing et al. [128], which compared TWS trends derived from five different GRACE solutions (JPL-SH (spherical harmonic), CSR-SH, GFZ-SH, JPL-M (mascon), and CSR-M), revealed a variation of up to 1.77 Gt/year for the entire TP, 2.88 Gt/year for the northwestern TP basins (the Tarim, Inner Tibetan Plateau, Qaidam, and Hexi basins), and 4.75 Gt/year for the southwestern TP basins (the Ganges, Brahmaputra, Salween, and Mekong basins). Long et al. [129] also noted that the signal-to-noise ratio in GRACE spherical harmonic solutions is particularly low over mountainous and complex terrains like the TP. To address this, they proposed using a spatially distributed scale factor from the WaterGAP Global Hydrology Model (WGHM) to correct bias and leakage errors in the GRACE retrievals. Several studies have focused on individual TWS components using specific methods such as observational analysis, modeling, or remote sensing (e.g., referring to studies in Section 3.1.2 that focus on glacier mass balance, lake storage changes, and permafrost freezing/thawing dynamics). Despite some consensus on individual storage changes, such as significant lake expansion over the TP with a more rapid rate in the northern plateau than the southern plateau [25], the results of these single-method approaches are not well constrained. Therefore, they fail to capture the full contribution of each storage component to the overall TWS change. To obtain a more accurate understanding of TWS changes over the TP, it is crucial to improve our understanding of individual storage components and climate drivers responsible for these changes.

Another limitation of GRACE data is the gap between the GRACE and GRACE-FO missions (July 2017–May 2018), which creates a temporal discontinuity in TWS analysis. During this gap, data-driven approaches, such as machine learning models, have emerged as valuable tools for reconstructing TWS signals. Machine learning models can efficiently capture non-linear relationships between input and output variables, with their applications in reconstructing GRACE data evolving from simpler models (e.g., autoregressive integrated moving average (ARIMA) and multiple linear regressions (MLR)) to more complex structures, including random forest (RF), support vector machine (SVM), artificial neural networks (ANN), deep neural networks (DNN), and convolutional neural networks (CNN) [[130], [131], [132], [133], [134], [135]].

**Fig. 5:**
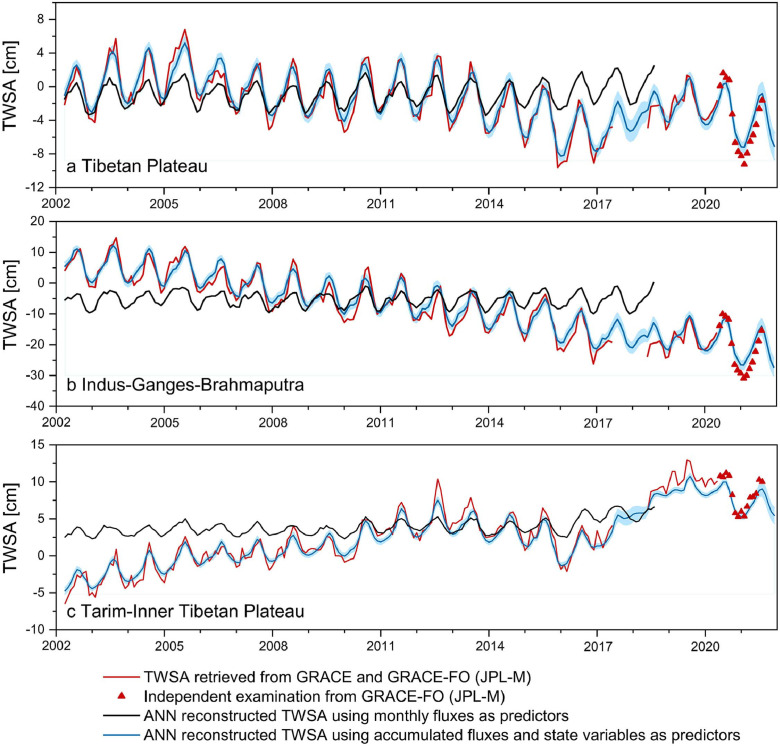
**Reconstructed TWS anomaly (TWSA) using artificial neural networks (ANN).** Region-averaged TWSA estimated from GRACE and GRACE-FO JPL-M solution and machine learning reconstructed outputs are presented during the 2002–2021 period over the Tibetan Plateau (a), Indus-Ganges-Brahmaputra (b), and Tarim-Inner Tibetan Plateau regions (c). Red lines show GRACE and GRACE-FO retrievals. Red triangles indicate GRACE-FO observations during Jun 2020–Aug 2021 that were excluded from training, validating, and testing samples of the machine learning models. Black lines show ANN-reconstructed TWSA using monthly fluxes as predictors, provided by Sun et al. [132]. Blue lines show ANN-reconstructed TWSA using accumulated fluxes and state variables as predictors, provided by Li et al. [3].

In reconstructing GRACE temporal signals, previous studies typically used predictors such as monthly precipitation, temperature, ET, soil moisture, and climate indices (e.g., sea surface temperature) [[131], [132], [133]]. While this approach can be effective in humid regions, it tends to produce poor results in arid and high-mountain regions like the TP. For instance, when comparing the ANN-reconstructed TWS anomalies [132] with GRACE-retrieved TWS anomalies over the TP, the reconstructed TWS change (represented by black lines in Fig. 5) does not show the expected trends (e.g., increasing TWS in the Tarim-Inner TP region and decreasing TWS in the Indus-Ganges-Brahmaputra region) when compared to GRACE observations (red lines and dots in Fig. 5). Additionally, caution is required when using complex machine learning models, as they are prone to overfitting, particularly with the relatively small sample size provided by GRACE and GRACE-FO data (∼240 months). It is crucial to perform thorough posterior validation of machine learning outputs, such as assessing the distributions, bias, and root mean square errors of residuals in testing datasets. Unfortunately, this step is often overlooked in many studies. In addition to reconstructing temporal signals, machine learning techniques have also been used to improve the spatial resolution of GRACE data by incorporating high-resolution climate and vegetation factors as input predictors [136,137]. However, specific studies on GRACE downscaling over the TP remain limited. As a result, more research is needed to explore the full potential of machine learning in improving GRACE-related studies, particularly over high-mountain regions like the TP.

#### 3.3 Tackling challenges in satellite retrievals of water storage changes

In this section, we explore potential strategies to enhance remotely sensed TWS change estimates over the TP, with an emphasis on integrating multisource remote sensing data and applying hydrologic principles to refine machine learning models.

One promising approach is the integration of multisource data from satellite gravimetry, altimetry, and optical remote sensing. By combining top-down (GRACE-based retrievals) and bottom-up (component-specific water storage) methodologies, we can establish a more robust benchmark for TWS changes. For instance, Li et al. [3] quantified bottom-up TWS changes over the TP by summing glacier mass loss (calculated from elevation changes provided by Brun et al. [21] and the glacier mask of RGI 6.0), lake storage variations (using datasets from Li et al. [23] and Wang et al. [138]), and subsurface water components (averaged from simulations by three land surface models). They then compared these bottom-up TWS change estimates with top-down TWS changes derived from four GRACE solutions (JPL-M, CSR-M, JPL-SH, and CSR-SH). Their analysis showed good agreement between the JPL-M retrievals and the bottom-up estimates for most TP regions. For example, JPL-M data revealed that climate change has caused severe TWS depletion in the TP’s exorheic basins (−15.8 Gt/year) but led to significant increases in TWS in the TP’s endorheic basins (5.6 Gt/year) during the 2002−2017 GRACE observation period.

By combining these top-down and bottom-up approaches, Li et al. [3] demonstrated that glacier mass loss accounted for approximately 76% of the TWS depletion in the Indus-Ganges-Brahmaputra basins, while lake water gain contributed about 137% of the TWS increase in the Inner TP basins. This agreement suggests that leveraging multisource remote sensing data is highly effective for quantifying TWS changes and assessing the contribution of individual water storage components. However, given the uncertainty associated with different datasets, we emphasize that this combined framework is better suited for cross-validation rather than direct calculation of individual storage components through mass balance. Previous studies have attempted to estimate groundwater storage, using the approach of subtracting remotely sensed surface water storage and model-based soil moisture changes from GRACE-derived TWS changes [92,139,140], or separating signals of individual storage components from TWS signals based on spherical harmonic expressions and mascon-fitting formulas [141]. However, it is crucial for these studies to account for uncertainty in each storage component and the propagation of uncertainty across different data sources; otherwise, the final estimates of storage change may be misleading.

Regarding machine learning-based TWS reconstruction, although these algorithms do not explicitly model physical processes, integrating hydrologic principles into the selection of predictors can significantly improve their performance. For example, Zhang et al. [91] found that accumulated standardized precipitation was more stronger linked to lake mass changes (cumulative water gain) than monthly precipitation, which aligns with the water balance principle (Input fluxes − Output fluxes = TWS changes). This finding suggests that TWS anomalies (the net change in TWS over a period) are better captured by accumulated fluxes rather than by monthly fluxes. Building on this principle, Li et al. [3] and Mo et al. [130] incorporated both accumulated inflows and outflows, as well as state variables, to develop non-linear relationships between input predictors and GRACE-retrieved TWS anomalies. These improvements led to more accurate TWS trend estimates compared to previous studies [132] (e.g., blue lines in Fig. 5).

Additionally, various statistical indices can be used to assess the risk of overfitting in machine learning models. These include bias, root mean square error (RMSE), and the distribution of residuals. A well-performing model should exhibit low bias, low RMSE, and residuals that are randomly distributed and close to a normal distribution. In contrast, an overfitted model will show low bias but high variance, along with systematically skewed residuals (either overestimated or underestimated). This suggests the importance of rigorous validation to ensure that machine learning models are robust and generalizable.

In conclusion, recent advancements in machine learning offer significant potential for hydrologic analysis. Future research can further harness this potential by integrating hydrologic insights into model design, using more physically guided input variables, and leveraging the larger datasets that are becoming available through satellite observations. By combining these strategies, machine learning models can play a crucial role in improving our understanding of water storage changes, particularly in regions like the TP.

## 4 Runoff estimation over major Asian rivers

Climate-induced changes in the hydrologic cycle, such as alterations in precipitation phase (the ratios of rainfall and snowfall to precipitation) and solid water storage, have significantly impacted runoff trends, extremes, seasonality, and variability, particularly over the TP. In-situ streamflow gauges are mostly located in the southern and eastern TP rivers (Fig. 2), including headwaters of the Brahmaputra, Salween, Mekong, Yangtze, and Yellow rivers. Observation-based runoff analysis over the TP mainly focuses on these five river basins, hereafter referred to as “China’s southwestern river basins”. Using statistical approaches like trend analysis and abrupt change detection, published studies have analyzed long-term runoff trends and shifts over this region. For example, Long et al. [19] examined long-term trends in runoff during 1981–2020 using streamflow observations of five gauges at the mainstream of China’s southwestern river basins. Xu et al. [142] included 19 gauges at both mainstreams and tributaries to examine variations in runoff from these river basins for the period 1950–2010. Tian et al. [143] analyzed runoff variations using 12 gauging stations from 1980 to 2015 in six major exoreic TP rivers, including China’s southwestern river basins and the Hei River basin. Zhang et al. [144] further extended the study period to the recent 6 decades (1960–2020) and examined variations in both streamflow and sediment. Although these studies covered different time periods, they generally found a slight runoff decrease in the Yellow River basin for the past 4–6 decades, whereas all other basins showed an increase in runoff. In addition, some studies also revealed regional disparities in runoff response to climate change, with the northern and southern parts of the TP exhibiting distinct trends [19,143]. Specifically, over the past 4 decades, a transition of runoff trends of China’s southwestern river basins was found around the year 2000. Southern basins witnessed an increasing trend before the year 2000, followed by a decline, while northern basins demonstrated an inverse trajectory in both periods. In general, runoff changes in China’s southwestern river basins are highly related to precipitation changes [19,142,143] and show strong seasonality with peaks occurring in the monsoon period (a unimodal distribution) [145]. Variations in the wet-dry period over this region were mostly attributed to El Niño–Southern Oscillation (ENSO) [142] and the westerly-monsoon circulation [19].

In contrast, changes in runoff result from the combined effects of precipitation and snow and glacier meltwater in the western higher-altitude TP. Solid precipitation (snowfall) at high elevations (e.g., the Himalayas) often freezes in the cold seasons, and there is a lag of several months before it melts into liquid water and translates into runoff as it gets warmer. Therefore, observed runoff could lag precipitation for several months, resulting in peak flows that differ from precipitation peaks. For example, Li et al. [50] indicated that precipitation over the Indus basin on the TP exhibits a bimodal annual distribution, where the two peaks originate from snowfall in spring and rainfall in summer. But there is only one peak of observed runoff, which occurs in July due to both large rainfall and meltwater.

So far, the long-term and continuous discharge records are available for only a few river basins. Runoff observations are predominantly available for lower-elevation regions of the southern and eastern TP, which limits a comprehensive understanding of runoff dynamics across the entire TP, a region characterized by significant spatial heterogeneity. The stringent data-sharing policies of transboundary rivers further exacerbate the scarcity of runoff data for observation-based analyses. Moreover, runoff changes on the TP are jointly influenced by precipitation (snowfall and rainfall), snow and glacier melt, and permafrost dynamics, which are challenging to directly quantify from field observations, particularly for large river basins. A more comprehensive understanding of runoff changes on the TP, including contributions of different runoff components to total runoff, the response of runoff changes to a warming climate, and hydro-climatic mechanisms underlying runoff changes, requires a combination of observed data, hydrologic and cryosphere modeling, and numerical simulations. This situation presents a key opportunity for the development of advanced modeling techniques, including data-driven and optimization-based algorithms, to improve the accuracy of runoff simulations.

### 4.1 Runoff simulation over the Tibetan Plateau

#### 4.1.1 Developments in hydrologic models

Hydrologic models are crucial tools for assessing runoff changes in poorly-gauged basins, such as those on the TP. Observed runoff data are generally essential for calibrating and validating the parameters of runoff models. Additionally, ancillary datasets, such as snow cover area, snow water equivalent, and TWS changes, are invaluable for improving our understanding of runoff processes, particularly in high-mountain regions like the TP [86,146,147]. Given the TP’s unique cryospheric characteristics, hydrologic models designed to simulate runoff in this region should incorporate modules for both glaciers and snow to accurately represent surface runoff processes driven by glaciers and snowmelt.

Two widely used approaches for quantifying meltwater are the degree-day melt algorithm [148] and the energy-balance model [149,150]. Several studies have successfully integrated these approaches into traditional hydrologic models. For example, Chen et al. [86] developed a snow and glacier module, CREST-Snow, based on the Coupled Routing and Excess STorage (CREST) model [151] to simulate both total runoff and snow/glacier melt over the Upper Brahmaputra River basin on the TP. Cui et al. [27] modified the Tsinghua Representative Elementary Watershed (THREW) model [152], adding modules for glacier evolution and snowpack simulation to improve its accuracy over the TP. Su et al. [153] augmented the Variable Infiltration Capacity (VIC) model [154,155] with a degree-day melt algorithm (VIC-glacier), using different degree-day factors for glaciers and snow, to quantify glacier and snowmelt in four western TP basins. Chai et al. [156] enhanced the three-layer energy balance snow module of the Water and Energy Budget-based Distributed biosphere Hydrologic Model (WEB-DHM), incorporating glaciers as snow with a thickness of 100 m to better simulate snow and glacier meltwater in the Salween River basin.

In addition to climate-induced changes in surface runoff, the freeze-thaw process of soil plays a crucial role in subsurface runoff generation over the TP. The region is home to the world’s largest alpine permafrost zone, which has undergone significant degradation over recent decades [[157], [158], [159]]. Warming-induced permafrost degradation generally leads to increased baseflow, faster groundwater recession, and an expanded groundwater storage capacity [160,161]. To model these processes, hydrologic models that couple heat and water transfer within frozen soils typically use either analytical or numerical methods [162,163].

The Stefan equation is a commonly applied analytical method that estimates the depth of frozen soil and the volume of frozen groundwater based on ground temperature and soil thermodynamic properties [[164], [165], [166]]. While this method requires relatively few data points, it generally neglects the impact of frozen soil on soil hydraulic properties. Numerical models, on the other hand, couple soil and groundwater dynamics in hydrologic and land surface models, using permafrost distribution data to simulate water content changes in thawing soil layers. For example, He et al. [167] used an integrated surface-subsurface model based on HydroGeoSphere (HGS) [168], incorporating a thin, low-permeability permafrost layer to quantify runoff variations in the Lhasa River basin. Yao et al. [169] developed a groundwater model based on MODFLOW-NWT [170] for the Upper Brahmaputra River basin, dividing the region into eight hydrogeological-permafrost units that simulate the seasonal transition from “aquitard” to “aquifer”. Additionally, baseflow separation methods, such as those based on recession curves and flow duration curves [171,172], are used to estimate subsurface flow. However, these empirical methods often rely on parameters that introduce considerable uncertainty in regions with complex soil properties.

As hydrologic models have evolved, recent research has increasingly focused on quantifying the contributions of various runoff components, such as rainfall runoff, meltwater, and groundwater, to total runoff over the TP, both historically and in future projections (e.g., Table 4). Changes in these runoff components can have significant downstream impacts, particularly on water resource management for sectors like agricultural irrigation [4,5,7] and on water scarcity driven by rising populations [33]. A key finding across recent studies is that the major rivers originating from the TP have experienced substantial changes in total runoff and runoff components since the 1980s [1]. Furthermore, future warming is expected to drive extreme runoff events that could exceed historical extremes [153,173,174]. However, the contributions of different runoff sources to total runoff change remain inconsistent in both past assessments and future projections (see Section 4.2).

**Table 4:** **A summary of recently published studies related to runoff simulation over the Tibetan Plateau**.

Previous study	Basin	Model	Number of gauges	Time period
Immerzeel et al. [175]	Indus, Ganges, Brahmaputra, Yangtze, Yellow	SRM	1	2000−2007; 2046−2065
Immerzeel et al. [176]	Indus, Ganges	Glacio-hydrological model	1	2000−2100
Lutz et al. [26]	Indus, Ganges, Brahmaputra, Salween, Mekong	SPHY	8	1998−2007; 2041−2050
Su et al. [174]	Indus, Brahmaputra, Salween, Mekong, Yangtze, Yellow	VIC	6	1971−2000; 2011−2070
Chen et al. [86]	Brahmaputra	CREST-Snow	1	2003−2014
Biemans et al. [4]	Indus, Ganges, Brahmaputra	SPHY	6	1981−2010
Han et al. [146]	Yangtze	CREST-Snow	1	2003−2014
Zhao et al. [177]	Brahmaputra, Salween, Mekong, Yangtze, Yellow	VIC	5	1971−2100
Lutz et al. [5]	Indus, Ganges, Brahmaputra	SPHY	6	1981−2010; 2071−2100

*Note* SRM, Snowmelt Runoff Model; SPHY, Spatial Processes in Hydrology; VIC, Variable Infiltration Capacity. CREST-Snow, Coupled Routing and Excess Storage Model-Snow. The number of gauges indicates how many streamflow gauges were used for model calibration. The time period indicates the study period that was analyzed in each paper.

#### 4.1.2 Data-driven approaches and hybrid models for runoff estimation

In addition to traditional hydrologic models based on physical processes and empirical knowledge, data-driven approaches have emerged as powerful alternatives, particularly for simulating complex, non-linear relationships between hydrologic variables. These approaches, which include machine learning methods and various mathematical and statistical algorithms, offer significant potential for improving runoff estimation.

One of the most widely used data-driven methods for runoff simulation is the Long Short-Term Memory (LSTM) network. LSTM models are well-suited for capturing inherent hydrologic patterns from sequential data due to their ability to learn temporal dependencies [178,179]. Building on the LSTM framework, recent studies have integrated other deep learning models to further enhance runoff estimation over the TP. For example, Li et al. [180] combined convolutional neural networks (CNN) with LSTM to optimize multiple tasks simultaneously within a single model. This multi-task training not only improves model efficiency but also reduces the risk of overfitting, while enhancing the model’s ability to represent various hydrologic processes. Ni et al. [181] adopted a hybrid approach by coupling the wavelet transform with CNN to extract temporal features, then incorporated these pre-processing techniques with LSTM to enhance the accuracy of monthly runoff estimation. These data-driven approaches have demonstrated good performance in runoff estimation over TP basins. However, one persistent challenge with these methods is their lack of interpretability, often referred to as the “black-box” nature of machine learning models. This limitation makes it difficult to fully understand the underlying mechanisms driving the model’s predictions.

Hybrid models, which combine process-based models with data-driven methods, offer a promising solution by leveraging the strengths of both approaches. These models integrate physical processes and hydrologic principles with the data-driven flexibility of machine learning, providing a more comprehensive framework for runoff simulation. For example, Li et al. [182] developed a semi-distributed hybrid model that uses the process-based EXP-Hydro model [183] as the foundation, while embedding neural networks (ENNs) to optimize model parameters. This hybrid approach combines the deep learning model’s ability to capture complex parameter relationships with the physical constraints imposed by the process-based model, offering improved accuracy and robustness.

Another hybrid approach is the Soil Moisture to Runoff (SM2R) model [50] that utilizes soil moisture dynamics for runoff simulation. In SM2R, parameters are determined through multi-dimensional mathematical optimization, guided by the physical principle of water balance. The model constrains outgoing fluxes, such as ET and runoff, using mathematical functions (e.g., hyperbolic tangent for ET and exponential forms for runoff), without requiring observed runoff for calibration. This approach builds on earlier work with the Soil Moisture to Rain (SM2RAIN) algorithm [184,185], which initially estimated precipitation and, subsequently, ET, drainage, and irrigation based on soil moisture [50,186,187]. The SM2R model has been applied to estimate runoff across 20 basins in the TP, outperforming both reanalysis outputs and 16 global hydrologic models. This highlights the potential of soil moisture-based hybrid models for runoff estimation, particularly in ungauged and data-sparse regions.

Compared to traditional hydrologic models, hybrid models offer several advantages. They leverage the strengths of mathematical optimization to systematically constrain storage and flux variables within physical limits, reducing the reliance on a large number of prior parameters. This results in a more efficient calibration process, with higher flexibility and improved capacity to derive optimal parameters.

### 4.2 Challenges in runoff estimation over the TP

Extrapolating information from gauged to ungauged or poorly gauged watersheds remains a long-standing challenge [36]. A significant limitation in accurate runoff estimation over the TP is the insufficient observation network, which is particularly problematic given the large heterogeneity in the complex hydrologic and climatic conditions of the region. Traditional hydrologic models, which rely heavily on structure and parameters calibrated using observed runoff [35,37], may not fully capture the unique challenges of the TP. For example, Immerzeel et al. [175] calibrated the parameters of the Snowmelt Runoff Model (SRM) using observed runoff at the Besham Qila station in the Indus basin, then transferred the parameters to simulate runoff in other major river basins such as the Ganges, Brahmaputra, Yangtze, and Yellow rivers. Another caution of runoff simulation over the TP lies in the uncertainty associated with the forcing fluxes, particularly in high-elevation regions. In these areas, ground observations/gridded estimates of fluxes often lack representativeness. For instance, Immerzeel et al. [47] indicated that in the upper Indus basin, the amount of precipitation required to sustain observed mass changes in large glacier systems far exceeds the values recorded at valley stations or those estimated by gridded precipitation products, sometimes by factors of two to 10. Similarly, Miao et al. [188] found considerable underestimation of precipitation over the TP when compared to estimates based on water balance considerations, which incorporated observations of ET, streamflow, and accumulated snow. Another complicating factor is the frozen soil process, which significantly increases the hydrologic complexity in cold regions. This is a crucial source of uncertainty in hydrologic model simulations and runoff component estimation. However, the lack of a comprehensive soil observation network across the TP severely limits our ability to validate and incorporate soil processes [189], further diminishing the reliability of subsurface runoff generation estimates.

Additionally, the risk of equifinality, where different parameter sets are equally plausible during model calibration and validation, remains a major challenge in runoff estimation over the TP [38,39]. Given the compensatory effects among different runoff sources in this region (e.g., a decrease in rainfall runoff may be offset by an increase in snowmelt), reliance on total discharge for model calibration requires more rigorous validation. Current models fail to adequately constrain the contributions of different water sources, leading to discrepancies in runoff component estimation. For example, studies on the Brahmaputra River’s source region (the Yarlung Zangbo River) report varying contributions of glacier meltwater to annual runoff, ranging from 5.5% [190] to 29% [191]. The contribution of groundwater runoff is even more uncertain, with estimates ranging from 4% [190] to 80% [192]. Projections of future runoff changes, which lack observational constraints, remain inconsistent across studies. For instance, Immerzeel et al. [175] projected reduced streamflow by the mid-21st century due to declining glacier meltwater in the Upper Indus, Ganges, and Brahmaputra basins, whereas Lutz et al. [26] predicted an increase in runoff throughout at least 2050, driven by increased precipitation (in the Upper Ganges and Brahmaputra) and accelerated glacier melt (in the Upper Indus). A recent projection [27] showed non-monotonic changes in river flow across seven rivers originating from the TP, in contrast to earlier studies that predicted a consistent increase in river flow [26,33,174].

The application of data-driven and hybrid models to runoff simulation over the TP remains in its infancy compared to the extensive use of physical models. Data limitations, as previously mentioned, hinder the development of robust relationships between key hydrologic variables. Furthermore, the “black-box” nature of data-driven models poses a challenge, as these models often fail to reveal the underlying mechanisms of hydrologic changes. For newly developed hybrid models, while they may show promising performance over selected basins, the integration of physical principles with data-driven approaches requires further refinement for broader application. For example, Feng et al. [193] indicated that combining deep learning models and hydrologic models as hybrid models could obtain differentiable, learnable, and process-based outputs without significantly reducing the deep learning skills for the target variable. However, this work has predominantly employed a lumped hydrologic model as the foundational framework in hybrid models, which fails to account for the spatially heterogeneous information of meteorological inputs and underlying surfaces. Li et al. [182] used a semi-distributed hydrologic model as the backbone to improve the spatial representation of deep learning neural networks, but the model performance has only been tested in three basins on the low-elevation eastern TP, which neglected critical cryospheric processes such as glacier melting and permafrost degradation. Another hybrid model, SM2R, simulates runoff based on the relationship between precipitation, runoff, and soil moisture at the monthly scale. Its ability to perform well at higher temporal resolutions, such as daily or hourly timescales, may be limited due to its structural limitations in capturing high-temporal-resolution runoff generation processes. Additionally, the SM2R model, being lumped in nature, represents basin-averaged characteristics and thus may struggle to capture spatially distributed variations in river flow. As soil data are fundamental to runoff estimation, the accuracy of SM2R could be compromised in regions with complex soil textures, such as the headwaters of the Yangtze and Yellow River basins, which exhibit signs of frozen soil degradation. To improve runoff estimations in permafrost regions, further research is needed to better understand the impacts of soil thawing and freezing on runoff processes and to enhance the accuracy of soil texture data.

### 4.3 Potential avenues for improving runoff estimation

Both advancements in observation networks and improvements to hydrologic models are crucial for overcoming the challenges associated with runoff estimation over the TP. While global observation networks like the Global Runoff Data Centre (GRDC) offer limited data for the TP, there are valuable local and project-specific databases that can provide essential data for refining runoff estimates. These include: (1) China’s Second Tibetan Plateau Scientific Expedition and Research (STEP), (2) the Chinese Academy of Sciences’ “Pan-Third Pole Environment Study for a Green Silk Road (Pan-TPE)” and “TP-River” project, (3) NASA’s High Mountain Asia Team (HiMAT), (4) HMA data from the National Snow and Ice Data Center (NSIDC), and (5) the International Centre for Integrated Mountain Development (ICIMOD). These projects provide critical in-situ data on glacier thickness, snow depth, river flows, and soil moisture, which are vital for understanding hydrologic and cryospheric processes across the TP. For instance, the “TP-River” project estimated that the annual total river runoff of 13 rivers originating from the TP was approximately 656 ± 23 km^3^ in 2018 [194].

In addition to in-situ data, emerging data sources such as isotopic measurements and remotely sensed data hold significant potential to complement the observation network and improve the accuracy of runoff simulations. Isotopic data, for example, can help diagnose model structure by revealing the influence of specific processes, such as frozen soil [195]. Tracer-aided hydrologic models have been shown to improve soil moisture simulation [196] and better delineate runoff sources [197]. While remote sensing cannot directly measure runoff, it can provide valuable ancillary information, including river widths, water levels, TWS changes, snow cover, and ET. This additional information can significantly enhance model calibration in various ways. For example, remotely sensed river widths and water levels can provide calibration data at “virtual stations”, referring to locations where satellite orbits intersect water bodies [87,147,198,199]. Furthermore, GRACE-derived TWS changes and MODIS-derived snow cover area are strongly related to glacier and snowmelt runoff in high-mountain regions, offering valuable data sources to calibrate runoff models for glaciers and snowpacks over the TP [86,146]. Remotely sensed ET data have also been used to refine the calibration of hydrologic models, improving predictions of daily and monthly runoff through a water balance approach [200]. These new data sources can help reduce the risk of parameter equifinality, where model parameters may compensate for one another (e.g., more snowmelt compensating for less rainfall runoff), and enhance the rationality of the hydrologic processes and their spatial representation.

Another avenue for improving runoff estimation accuracy lies in developing a more comprehensive validation framework that accounts for variations in climate conditions, land cover types, and soil textures across the TP. This framework should include adjusting precipitation-runoff relationships for different land cover types, identifying dominant climate factors in monsoon- and westerly-controlled regions, and strengthening the link between surface and subsurface flows in areas with significant permafrost depletion. Such improvements would also help clarify the role of soil moisture in regulating precipitation-runoff relationships under various climate and land cover scenarios, an aspect that is often underestimated in current Earth System Models [[201], [202], [203]]. By leveraging these new observation sources and refining model algorithms, it may be possible to simulate spatially distributed runoff with high temporal resolution and accuracy across the TP, while also quantifying the contributions of different runoff components to total runoff.

## 5 Discussion and conclusion

This review highlights the strengths of newly developed observation techniques and interdisciplinary approaches in estimating changes in water storage and runoff over the TP. For water storage analysis, we summarize recent advancements in satellite gravimetry, altimetry, and optical remote sensing for estimating variations in lake storage, glacier mass, and TWS, as well as the successful application of machine learning models for reconstructing TWS changes. A major challenge in TWS change estimation from GRACE and GRACE-FO satellite missions is their relatively coarse spatial resolution (∼300 km), which limits their effectiveness over the highly heterogeneous TP. Based on the variation of different GRACE solutions (three spherical harmonic solutions of CSR, GFZ, and JPL and two mascon solutions of CSR and JPL), the uncertainty in TWS change over the TP during the GRACE period of 2002–2017 was estimated up to 1.77 Gt/year (= km^3^/year), with 2.88 Gt/year and 4.75 Gt/year for the northwestern TP basins and southwestern TP basins, respectively [128]. Additionally, the gap between the GRACE and GRACE-FO missions disrupts temporal continuity in TWS analysis. Hydrologic and land surface models tend to underestimate TWS trends due to the absence of explicit glacier and groundwater components, highlighting the advantages of data-driven machine learning methods in capturing non-linear hydrologic relationships. However, the complex interactions among hydrologic fluxes and storage components remain a key challenge in applying machine learning to reconstruct TWS change.

For runoff estimation, despite a broader body of research compared to water storage, traditional runoff models over the TP require further improvements to address challenges such as sparse observational data for model calibration, parameter equifinality, and large uncertainties in model inputs. Cui et al. [27] estimated that the uncertainty in daily runoff simulation, represented by the percent bias of simulation results relative to observed streamflow, was 9.7% (Indus), 5.4% (Ganges), −0.1% (Brahmaputra), 4.3% (Salween), 4.4% (Mekong), 5.3% (Yangtze), and 5.2% (Yellow) during the calibration period. For the validation period, the percent bias across these basins was −9.8% (Indus), −0.8% (Ganges), 0.6% (Brahmaputra), −3.0% (Salween), 1.6% (Mekong), −4.7% (Yangtze), and 12.8% (Yellow). In addition to the simulation uncertainty of total runoff, a reliable hydrologic process is critical to understand climate change impacts on the TP and to develop sustainable water management strategies. Given the compensatory effects among different runoff sources in this region (e.g., a decrease in rainfall runoff may be offset by an increase in snowmelt), reliance on total discharge for model calibration requires more rigorous validation for a better understanding of runoff component contributions. Published studies on the Brahmaputra River’s source region (the Yarlung Zangbo River) report varying contributions of glacier meltwater to annual runoff, ranging from 5.5% [190] to 29% [191], with an even more uncertain contribution of groundwater runoff ranging from 4% [190] to 80% [192]. New data-driven and hybrid modeling approaches have demonstrated promising results in selected basins, improving model optimization and runoff estimation capabilities. However, these approaches still require extensive validation to achieve high-temporal and spatially distributed runoff estimates under diverse climatic and land cover conditions.

To address these challenges, we suggest the following four key strategies. First, combining satellite gravimetry, altimetry, and optical images can enhance the accuracy of TWS change and storage component estimates, improving our understanding of the contributions of individual storage components to overall TWS changes. Second, while machine learning holds great promise, we emphasize the importance of integrating hydrologic and physical principles in model design to prevent overfitting and improve model interpretability. Selecting predictors based on hydrologic relevance rather than purely statistical relationships can enhance model robustness. Third, using observed data from local and project-specific programs, as well as isotope and remotely sensed datasets, can strengthen model calibration and improve runoff estimation accuracy. Fourth, further efforts are needed to refine hybrid models, including adjusting precipitation-runoff relationships for different land cover types, improving parameterization schemes, and expanding validation across diverse climatic zones.

Changes in water storage and runoff profoundly influence both the stability of water resources on the TP and water supply for downstream regions, which are critical to policymakers for sustainable water management. Combining multisource remote sensing and machine learning approaches, illustrated in Section 3, the entire TP is projected to experience a net loss of TWS of about 230 Gt by the mid-21st century (2031–2060) relative to an early 21st-century (2002–2030) baseline under the mid-range emissions scenario [3]. The loss of solid-water resources represents a major threat to water management over the TP, particularly over the Amu Darya and Indus basins, where future precipitation would not change much, but temperature is projected to rise significantly. Relative to the multi-year average water demand of downstream regions, the decline in upstream supply capacity caused by TWS loss could be up to about 120% and 80% of the downstream water-demand baseline in the Amu Darya and Indus basins, respectively [3]. This indicates that more alternative water-supply sources, such as water transfer projects, may be necessary to meet the amplified water shortage in the future, highlighting an improved water policy for these transboundary river basins.

Using hydrologic models mentioned in Section 4.1, a strong and increasing dependence of downstream irrigation on upstream meltwater was found over the Indus and Ganges basins of the TP [4,5]. Considering the accelerating loss of upstream glaciers and snowpack, environmentally sustainable management of multi-purpose reservoirs may be necessary to increase the region’s water-buffering capacity and relieve pressure on meltwater [5]. In addition, under a warming scenario of 1.5–4 °C above pre-industrial levels by the end of the 21st century, runoff is projected to increase about 1.0%–7.2% for seven exorheic rivers on the TP [33]. However, because population projections diverge across basins, this runoff increase is likely to reduce the population fraction living under water scarcity conditions in the Yangtze and Yellow basins but not in the Indus and Ganges basins. Based on analysis from both TWS changes and runoff simulation, we find that the highly-glacierized western TP basins (the Amu Darya, Indus, and Ganges basins) are generally more vulnerable than the eastern basins (the Yangtze and Yellow basins) [1]. In addition, the western TP is the origin of major transboundary rivers in Asia, and therefore, we highlight more efficient adaptation strategies of transboundary cooperation and conjunctive water use, considering upstream-downstream linkages over these vulnerable basins.

Overall, climate change has significantly altered the spatiotemporal dynamics of TWS and runoff over recent decades, yet observational and modeling limitations continue to hinder our understanding of terrestrial water system responses. This review synthesizes recent progress in retrievals and simulations of TWS change and runoff over the TP, highlighting advancements in observation techniques, data-driven approaches, and hybrid modeling strategies. These developments have important implications not only for the scientific community in understanding cryospheric and hydrologic responses to climate change, but also for policymakers in formulating adaptive water resource management strategies for this vulnerable high-mountain region.

## Data availability

Shapefiles of boundaries of the TP and hydrologic basins are available at http://data.tpdc.ac.cn. Locations of meteorological gauges from the China Meteorological Administration are provided at https://data.cma.cn/. Locations of GNSS stations from the Crustal Movement Observation Network of China are accessed at https://data.earthquake.cn/datashare/report.shtml?PAGEID=siteInfo_jizhun. Locations of streamflow gauges from the Ministry of Water Resources of the People’s Republic of China are provided at https://www.mwr.gov.cn/en. Precipitation and temperature data from China meteorological forcing dataset v2.0 [204] are available at https://doi.org/10.11888/Atmos.tpdc.302088.

## CRediT authorship contribution statement

**Xueying Li:** Writing – review & editing, Writing – original draft, Formal analysis, Data curation, Visualization. **Di Long:** Writing – review & editing, Supervision, Funding acquisition, Conceptualization, Data curation. **Bridget R. Scanlon:** Writing – review & editing. **Louise J. Slater:** Writing – review & editing.

## Declaration of competing interest

The authors declare that they have no conflicts of interest in this work.
